# Activation of protein phosphatase 2A in FLT3+ acute myeloid leukemia cells enhances the cytotoxicity of FLT3 tyrosine kinase inhibitors

**DOI:** 10.18632/oncotarget.10167

**Published:** 2016-06-18

**Authors:** Amanda M. Smith, Matthew D. Dun, Erwin M. Lee, Celeste Harrison, Richard Kahl, Hayley Flanagan, Nikita Panicker, Baratali Mashkani, Anthony S. Don, Jonathan Morris, Hamish Toop, Richard B. Lock, Jason A. Powell, Daniel Thomas, Mark A. Guthridge, Andrew Moore, Leonie K. Ashman, Kathryn A. Skelding, Anoop Enjeti, Nicole M. Verrills

**Affiliations:** ^1^ School of Biomedical Sciences and Pharmacy, University of Newcastle, Callaghan, New South Wales, Australia; ^2^ Hunter Medical Research Institute, Newcastle, New South Wales, Australia; ^3^ Current address: The University of Queensland Diamantina Institute, Woolloongabba, Queensland, Australia; ^4^ Children's Cancer Institute Australia for Medical Research, Lowy Cancer Research Centre, UNSW, Sydney, New South Wales, Australia; ^5^ Current address: Department of Medical Biochemistry, School of Medicine, Mashhad University of Medical Sciences, Mashhad, Iran; ^6^ Prince of Wales Clinical School, University of New South Wales, Sydney, New South Wales, Australia; ^7^ School of Chemistry, University of New South Wales, Sydney, New South Wales, Australia; ^8^ Centre for Cancer Biology, SA Pathology, Adelaide, South Australia, Australia; ^9^ Stanford Institute for Stem Cell Biology and Regenerative Medicine, Stanford University School of Medicine, Stanford, California, USA; ^10^ Department Clinical Haematology, Australian Centre for Blood Diseases, Monash University, Melbourne, Victoria, Australia; ^11^ Translational Research Institute, The University of Queensland Diamantina Institute, Woolloongabba, Queensland, Australia; ^12^ Calvary Mater Hospital, Newcastle, New South Wales, Australia

**Keywords:** PP2A, FTY720, AML, FLT3, tyrosine kinase inhibitor

## Abstract

Constitutive activation of the receptor tyrosine kinase Fms-like tyrosine kinase 3 (FLT3), via co-expression of its ligand or by genetic mutation, is common in acute myeloid leukemia (AML). In this study we show that FLT3 activation inhibits the activity of the tumor suppressor, protein phosphatase 2A (PP2A). Using BaF3 cells transduced with wildtype or mutant FLT3, we show that FLT3-induced PP2A inhibition sensitizes cells to the pharmacological PP2A activators, FTY720 and AAL(S). FTY720 and AAL(S) induced cell death and inhibited colony formation of FLT3 activated cells. Furthermore, PP2A activators reduced the phosphorylation of ERK and AKT, downstream targets shared by both FLT3 and PP2A, in FLT3/ITD^+^ BaF3 and MV4-11 cell lines. PP2A activity was lower in primary human bone marrow derived AML blasts compared to normal bone marrow, with blasts from FLT3-ITD patients displaying lower PP2A activity than WT-FLT3 blasts. Reduced PP2A activity was associated with hyperphosphorylation of the PP2A catalytic subunit, and reduced expression of PP2A structural and regulatory subunits. AML patient blasts were also sensitive to cell death induced by FTY720 and AAL(S), but these compounds had minimal effect on normal CD34+ bone marrow derived monocytes. Finally, PP2A activating compounds displayed synergistic effects when used in combination with tyrosine kinase inhibitors in FLT3-ITD^+^ cells. A combination of Sorafenib and FTY720 was also synergistic in the presence of a protective stromal microenvironment. Thus combining a PP2A activating compound and a FLT3 inhibitor may be a novel therapeutic approach for treating AML.

## INTRODUCTION

Fms-like tyrosine kinase 3 (FLT3; CD135) is a member of the type III transmembrane receptor tyrosine kinase family, together with PDGFR, c-KIT and c-FMS. FLT3 functions to promote cell survival and proliferation via activation of the MAPK, PI3K, and STAT5 signaling pathways [1]. FLT3 is expressed in normal hematopoietic progenitor cells, in most acute myeloid leukemia's (AML), and a smaller subset of B-cell acute lymphoblastic leukemia (ALL), blast crisis chronic myeloid leukemia (CML) and T-cell ALL [2]. Furthermore, AML cells frequently co-express FLT3 and its ligand (FL) establishing an autocrine or paracrine signaling loop resulting in constitutive FLT3 signaling [3]. In addition, mutations in FLT3 are the most common genetic mutation identified in AML. Internal tandem duplication (ITD), within the juxtamembrane domain of the receptor, occurs in approximately 24% of adult AML patients [4]. Point mutations within the activation loop of the kinase domain have further been reported in up to 7% of AML and 3% of ALL cases [4]. Both types of mutation result in constitutive activation of FLT3 tyrosine kinase activity and subsequent hyperactivation of its downstream signaling pathways [1]. Further, differential subcellular localization of the FLT3-ITD receptor within the perinuclear region results in interaction with, and activation of intracellular signaling proteins including ERK, STAT5 and AKT, not directly associated with the FLT3-WT receptor [5, 6]. The presence of FLT3 mutations confers a poor prognosis in AML, correlating with higher blast count and decreased remission induction rate, disease free survival, event free survival and overall survival [7].

FLT3 mutations function as oncogenic drivers in both mouse models of leukemia and human AML cells [8, 9], and therefore represent important therapeutic targets. Several tyrosine kinase inhibitors (TKIs) targeting FLT3 have been investigated in clinical trials, including CEP-701, PKC412, sorafenib and AC220. As single agents in AML trials these were disappointing with short-term and/or partial remissions being reported in a minority of patients [10]. However recent trials report improved survival in patients < 60 yrs treated with sorafenib [11] or PKC412 [12] in combination with chemotherapy. An emerging theme from clinical trials is that monotherapies targeting oncogenic kinases such as FLT3 lead to the selection of drug-resistant malignant clones and disease relapse [13, 14]. Thus, combination treatments that not only target oncogenic FLT3, but also its downstream signaling, such as the MAPK, PI3K and STAT5 pathways, may afford improved therapeutic responses in AML patients.

PP2A is a serine/threonine phosphatase that has emerged as an important tumor suppressor [15]. PP2A is a multimeric family of enzymes each composed of a catalytic (C), a scaffold (A), and one of a number of regulatory subunits (B/B55/PR55, B'/B56/PR56/PR61, B′/PR48/PR72/130, B″/PR93/PR110) that direct subcellular localization and substrate specificity (for a comprehensive review of PP2A structure and functions see [16, 17]). PP2A is further regulated by posttranslational modification and by endogenous interacting proteins such as SET and CIP2A [18]. The tumor suppressor activities of PP2A depend on its ability to inactivate multiple components of growth and survival signaling pathways required for tumorigenesis [18–20]. Functional inactivation of PP2A occurs downstream of BCR/ABL in CML and Ph^+^ ALL, and is essential for BCR/ABL induced leukemogenesis [21, 22]. Our previous studies have shown that the D816V oncogenic form of c-KIT requires inhibition of PP2A to promote leukemogenesis [23]. Importantly, pharmacological re-activation of PP2A not only inhibited proliferation and survival of D816V-AML cells *in vitro*, but also reduced their growth *in vivo* [23]. Impaired PP2A activity was further reported as a common event in AML, with 29/37 cases displaying inactivation [24], suggesting that AML sub-types without c-KIT mutations are also likely to exhibit PP2A inhibition. Indeed, in this study 6/7 FLT3-ITD patients displayed PP2A inhibition associated with altered PP2A subunit and/or SET expression [24]. As the c-KIT and FLT3 receptors are closely related and signal via similar downstream pathways [1], we hypothesized that PP2A may be inhibited downstream of FLT3 in AML, and hence therapeutic approaches that allow PP2A re-activation may have clinical benefit.

Herein, we show that activated FLT3 inhibits PP2A activity. Pharmacological activation of PP2A inhibited FLT3-mediated growth and survival of AML cells, and was synergistic with FLT3 TKIs. Given the high frequency of FLT3 activation and mutation in AML, these data suggest that PP2A activation may be a therapeutic strategy in the treatment of FLT3 driven malignancies.

## RESULTS

### Activation of FLT3 inhibits PP2A and sensitizes to PP2A activating drugs

The BaF3 cells are an established and very well characterised model for studying the molecular and functional consequences of oncogenic FLT3 signaling [25]. To investigate if activation of FLT3 regulates PP2A activity we stably transduced BaF3 cells with an empty vector (EV) or vectors containing the wildtype (WT) human FLT3 gene, or human AML-associated kinase domain mutations FLT3-D835V and D835Y, or FLT3 with an internal tandem duplication, FLT3-ITD. Surface expression of FLT3 was routinely monitored by flow cytometry (Supplementary Figure S1A). As expected, EV and BaF3/WT-FLT3 cells remained factor dependent. BaF3/WT-FLT3 could proliferate in the presence of either IL3 or FL, however their growth rate was slightly slower in FL as has been previously reported [26] (Supplementary Figure S1B–S1C). In contrast, expression of both of the FLT3-D835 mutants or FLT3-ITD, induced factor independent growth (Supplementary Figure S1B).

**Figure 1:**
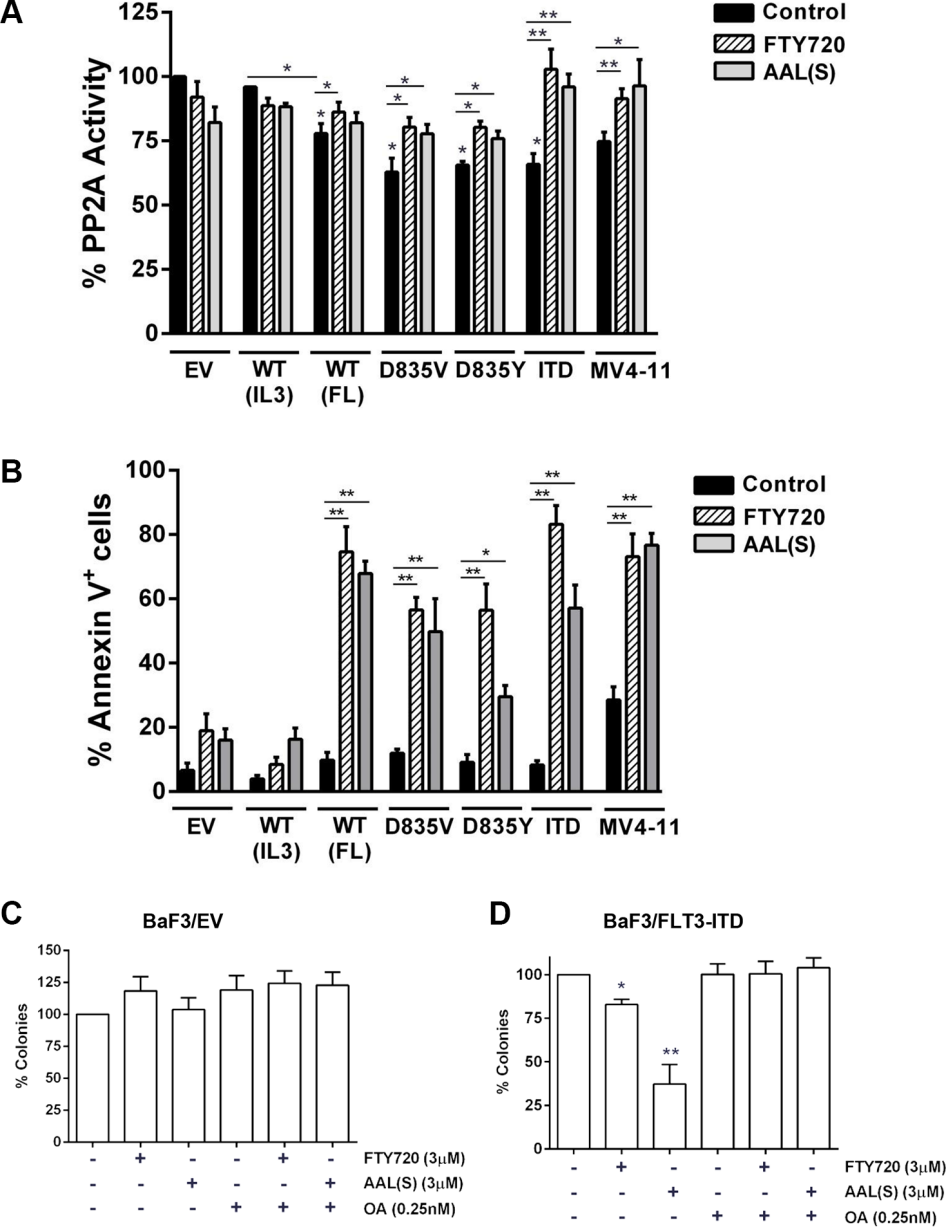
FLT3 activation inhibits PP2A and sensitizes to PP2A activating drugs (**A**) PP2A complexes were isolated from BaF3 or MV4-11 cells, treated with or without 3 μM FTY720 or AAL(S) for 12 h, using immunoprecipitation with an anti-PP2A-C antibody. PP2A activity was determined by incubating the isolated PP2A-C complex with a PP2A-specific phosphopeptide and measuring free phosphate released using a colorimetric assay. Activity was calculated as a percentage of control by dividing the activity of FLT3 transduced cells by untreated BaF3 empty vector (EV) controls. *Columns,* mean; *bars*, SEM. **p < 0.01, Student's *t* test compared with EV control. (**B**) Cells were treated with 5 μM (BaF3 cell lines) or 2 μM (MV4-11 cells) FTY720 or AAL(S) for 24 hr and apoptosis determined by annexin V^+^ staining and flow cytometry. *Columns;* mean, *bars;* SEM, *p < 0.05, **p < 0.01 compared to EV or untreated cells, as indicated; Students *t* test. (**C**) BaF3/EV and (**D**) BaF3/FLT3-ITD cells were grown in methylcellulose medium for 7 days in the presence of 3 μM FTY720 or AAL(S) ± 0.25 nM okadaic acid (OA). *Columns*, mean colony number (*n* = 3) relative to untreated; *bars*, SEM. *p < 0.05, **p < 0.01 compared to untreated cells.

We measured the phosphatase activity of PP2A immune-complexes isolated from the BaF3 cells. Activation of FLT3 with FL significantly reduced PP2A activity (78%) compared to EV cells (100%) or FLT3-WT cells grown in IL3 (96%) (Figure 1A). Constitutive activation of FLT3 by oncogenic mutation also significantly inhibited PP2A activity, with FLT3-D835V displaying 63%, FLT3-D835Y 66%, and FLT3-ITD 66% activity compared to EV cells (Figure 1A). Therefore activation of FLT3 inhibits PP2A activity. Interestingly, while PP2A enzyme activity was decreased, this did not correlate with a change in phosphorylation of PP2A-C (Y307) (Supplementary Figure S2A), indicating an alternative mechanism of enzyme inhibition in these cells.

Previous studies show that leukemia cells with low PP2A activity are sensitive to cell death induced by the pharmacological PP2A activator, FTY720 [21, 23, 27]. To determine if activation of FLT3 affected sensitivity to FTY720 we first examined the effect on PP2A phosphatase activity. FTY720 (3 μM; 12 h) increased PP2A activity in all cells signaling through FLT3, with the most striking increase in the FLT3-ITD cells (Figure 1A). In contrast FTY720 had no significant effect on PP2A activity in the EV or WT-FLT3 cells in IL3. (Figure 1A). Consequently, FLT3^+^ cells were more sensitive to inhibition of proliferation by FTY720 with lower IC50 values compared to control cells (Table 1). FTY720 is phosphorylated *in vivo* by sphingosine kinase-2 to form FTY720-phosphate (FTY720-P) [28, 29]. FTY720-P acts as a functional antagonist of the sphingosine-1-phosphate receptor mediated signaling pathway [30, 31]. To establish whether the cytotoxic effect of FTY720 depended on PP2A activation or sphingosine-1-phosphate receptor antagonism, we utilized an analogue of FTY720, AAL(S), that cannot be phosphorylated by sphingosine kinase-2, but can still activate PP2A [32]. AAL(S) activated PP2A (Figure 1A) and reduced the viability of FLT3^+^ cells to a similar extent as FTY720 (Table 1). This was confirmed in an independent cell line, the FDC.P1 mouse myeloid progenitor cells [33] expressing WT-FLT3 (Supplementary Figure S1D–S1E; Table 1). We further examined the effect of PP2A activators in the human FLT3-ITD^+^ AML cell line, MV4-11. FTY720 and AAL(S) (3 μM, 12 hr) significantly enhanced PP2A activity (Figure 1A), and induced growth inhibition (Table 1) of MV4-11 cells.

**Table 1:** Growth inhibition by FTY720 and AAL(S)

Cell Line	IC50 FTY720 (μM)^1^	IC50 AAL(S) (μM)^1^
BaF3/EV	7.1 ± 0.3	7.9 ± 0.3
BaF3/WT + IL3	6.9 ± 0.3	8.5 ± 0.3
BaF3/WT + FL	3.7 ± 0.2** ^§§^	5.3 ± 0.6** ^§§^
BaF3/D835V	5.2 ± 0.2**	5.2 ± 0.2**
BaF3/D835Y	5.3 ± 0.3**	5.3 ± 0.2**
BaF3/ITD	4.0 ± 0.3**	4.6 ± 0.7**
FDC.P1/WT + GM	5.1 ± 0.2	5.4 ± 0.0
FDC.P1/WT + FL	2.7 ± 0.3^^^^	3.1 ± 0.3^^^
MV4-11 (ITD)	3.6 ± 0.3	3.8 ± 0.3

1 IC50 is the concentration (μM) of drug required to reduce cell viability by 50% at 48 h and was calculated using fit-spline/cubic regression. Data are presented as the mean of at least three independent experiments performed in triplicate ± SEM.

** *p* < 0.01 compared to BaF3/EV;

§§ *p* < 0.01 compared to BaF3/WT+IL3;

^ *p* < 0.05

^^ *p* < 0.01 compared to FDC.P1/WT+GM.

Next we examined cell death induced by FTY720 and AAL(S). Treatment of cells with 5 μM FTY720 or AAL(S) for 24 h induced significant apoptosis in the BaF3/FLT3^+^ cells, as assessed by Annexin V^+^ staining, but had minimal effects on control cells (Figure 1B). FTY720 and AAL(S) both induced similar levels of apoptosis in WT-FLT3 cells in the presence of FL, and in the FLT3-D835Y cells, but AAL(S) was slightly less effective than FTY720 at inducing apoptosis in the FLT3-ITD and FLT3-D835V cells. The MV4-11 cells were remarkably sensitive to apoptosis induction with FTY720 and AAL(S), with 100% cell death at 5 μM of either drug (not shown), and 73% and 77% Annexin V^+^ cells with 2 μM FTY720 and AAL(S), respectively (Figure 1B). Finally, we showed that the apoptosis induced by FTY720 requires PP2A activation, as the PP2A inhibitor, okadaic acid, rescued the effects in BaF3/FLT3-ITD and MV4-11 cells (Supplementary Figure S3). Furthermore, colony formation of the BaF3/FLT3-ITD cells was modestly inhibited by FTY720, and more profoundly inhibited by AAL(S), and addition of OA restored colony formation in the presence of either drug (Figure 1D), confirming that the colony inhibition induced by these drugs was due to PP2A activation. In contrast, FTY720 or AAL(S) had no effect on clonogenicity in the BaF3/EV cells (Figure 1C). Together this data shows that re-activation of PP2A with two independent compounds reduces the viability of cells signalling through FLT3, and the mechanism of action does not require sphingosine-1-phosphate receptor inhibition.

### PP2A inhibition is associated with reduced expression of PP2A subunits and is dependent on FLT3-ITD activation

**Figure 2:**
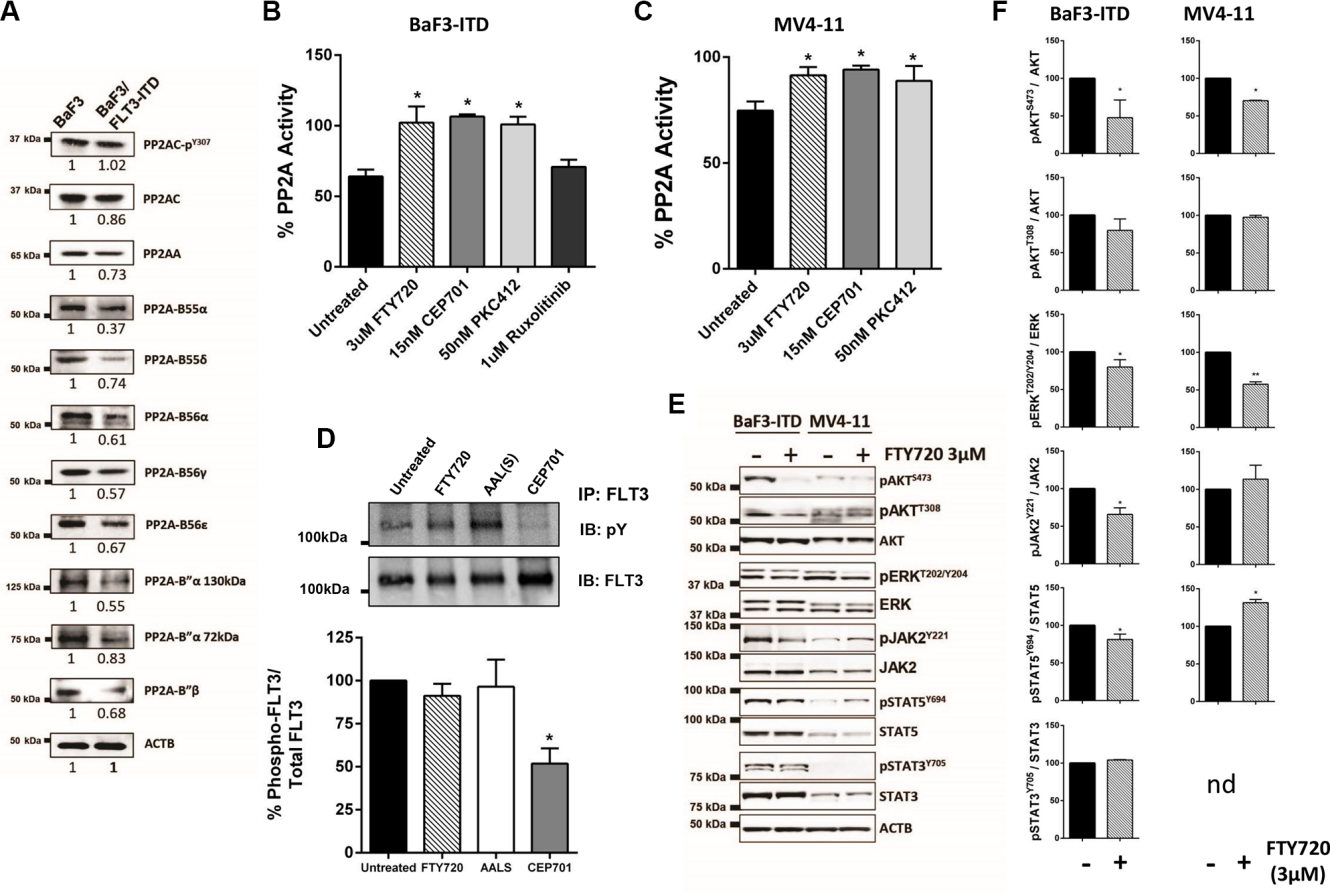
FLT3-ITD regulates PP2A activity and expression, and PP2A re-activation inhibits FLT3 downstream signaling (**A**) Immunoblots of BaF3 cell lysates showing expression of PP2A-A, PP2A-C, p^Y307^PP2A-C, -B55α, -B55δ, -B56α, -B56γ, -B56δ, -B56ε, -B”α (130 kDa and 72 kDa) and B”β. β-Actin (ACTB) was used as a loading control. Blots are representative of at least four independent experiments. Values underneath each blot represent the mean expression ratio (relative to BaF3 cells) determined by dividing the densitometric volume of the test band by that of the actin band (see Supplementary Figure S2B). (**B** and **C**) PP2A activity was determined as in Figure 1 in B) BaF3/FLT3-ITD and C) MV4-11 cells treated for 12 hr with the indicated drugs. *Columns*, mean; *bars*, SEM. **p < 0.01, Student's *t* test compared with parental control. (**D**) FLT3 was isolated from BaF3/FLT3-ITD cells by immunoprecipitation followed by western blot after treatment with FTY720 (3 μM), AAL(S) (3 μM) or CEP701 (5 nM) for 24 h. The top blot shows total phosphotyrosine levels and the bottom total FLT3 levels. The densitometric volume of the phosphotyrosine was divided by that of the total FLT3 bands, and shown as a percentage relative to untreated cells. *Columns*, mean (*n* = 3); *bars,* SEM, *p < 0.05. (**E**) Immunoblots detecting phosphorylated and total levels of pAKT^S473^, pAKT^T308^ and total AKT, pERK1/2^T202/Y204^/ERK1/2, pJAK2^Y221^/JAK2, pSTAT5^Y694^/STAT5 and pSTAT3^Y705^/STAT3 in BaF3/FLT3-ITD and MV4-11 cells. β-Actin (ACTB) was used as a loading control. Images are representative of 3 independent experiments. (**F**) The relative expression ratio for each protein shown in (E) was determined by dividing the densitometric volume of the phosphorylated band, by that of the total band, and normalized to untreated cells. *Columns* mean expression, *Bars* SEM, ^*^p < 0.05 Students *t*-tests compared to untreated cells. nd; not detectable.

To investigate the mechanism by which PP2A is regulated by FLT3, we focused on cells expressing the most common AML-associated FLT3 mutation, FLT3-ITD. Firstly, we examined the expression of PP2A subunits in the BaF3/FLT3-ITD cells. Total levels of PP2A-C were slightly reduced in the FLT3-ITD cells compared to control cells, however there was no significant change in Y307 phosphorylation of PP2A-C (Figure 2A, Supplementary Figure S2A, S2B). The scaffolding PP2A-A subunit was significantly reduced in BaF3/FLT3-ITD cells relative to control cells. Furthermore, PP2A-B55α, –B55δ, -B56α, -B56γ, -B56ε, -B”α (130 kDa), -B”α (72 kDa) and -B”β (48 kDa) protein levels were all significantly lower in FLT3-ITD cells (Figure 2A, Supplementary Figure S2B). Thus, FLT3-ITD expression is associated with reduced expression of PP2A subunits together with an overall reduction in PP2A activity in BaF3 cells.

Next we examined whether pharmacological inhibition of FLT3 affected PP2A activity. Treatment of BaF3/FLT3-ITD cells with CEP701 or PKC412 resulted in a significant increase in PP2A activity, comparable to that induced by FTY720 (Figure 2B). Similar effects were observed in MV4-11 cells (Figure 2C). This suggests that the PP2A inhibition is dependent on FLT3-ITD activation. PP2A activity has previously been reported to be regulated by JAK2 [34], and expression of FLT3-ITD induced phosphorylation of JAK2 (Supplementary Figure S2C). Therefore, we further tested whether the PP2A inhibition was downstream of JAK2. In contrast to FLT3 inhibition, the JAK1/2 inhibitor Ruxolitinib had no effect on PP2A activity (Figure 2B), suggesting that the PP2A inhibition in these cells is not dependent on JAK2.

### Activating PP2A inhibits FLT3-ITD signaling downstream of FLT3

FLT3 activity is regulated by autophosphorylation of tyrosine residues, and previous studies have shown that FTY720-induced PP2A activation results in reduced phosphorylation of BCR/Abl [21] and c-KIT [23]. FTY720 and AAL(S) had no effect on phosphorylation of the FLT3-ITD receptor (Figure 2D). In contrast, the FLT3 inhibitor CEP701 significantly reduced FLT3-ITD phosphorylation (Figure 2D). This suggests that the effects of FTY720 and AAL(S) are mediated downstream of FLT3-ITD receptor activity. FLT3-ITD induced phosphorylation of JAK2, p38 MAPK, ERK, and AKT (Supplementary Figure S2C). FTY720 caused a significant reduction in phosphorylation of AKT(S473) in both the BaF3/FLT3-ITD and MV4-11 cells, but no significant change in phosphorylation of AKT at T308 (Figure 2E–2F). pERK was also significantly reduced with FTY720 treatment in both cell lines. pJAK2(Y221) was reduced in the BaF3/FLT3-ITD cells, with a concomitant decrease in pSTAT5(Y694). In contrast, FTY720 had no effect on pJAK2(Y221), and increased pSTAT5(Y694) in the MV4-11 cells. FTY720 had no effect on pSTAT3(Y705) in either cell line (Figure 2E–2F). Despite the increase in PP2A activity, we observed no change in phosphorylation of PP2A-C (Y307) with FTY720 treatment in the MV4-11 or BaF3/FLT3-ITD cells (Supplementary Figure S2A).

### Low PP2A activity in leukemic blasts from AML patients

**Figure 3:**
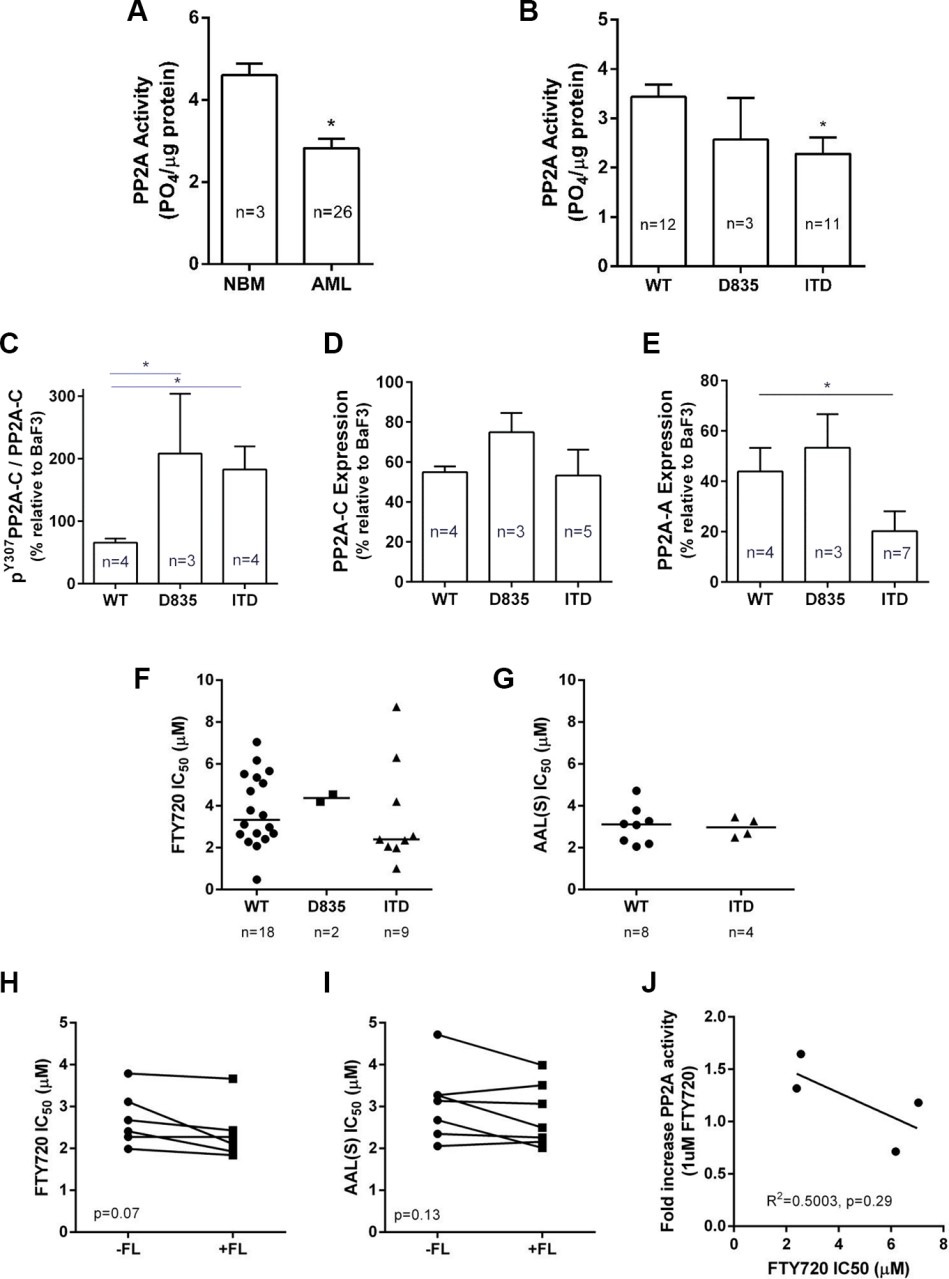
PP2A activity, expression and drug sensitivity in human AML mononuclear cells (**A**) Bone marrow mononuclear cells were isolated from healthy individuals (normal bone marrow; NBM) or AML patients by Ficoll gradient separation, and PP2A activity determined by immunopurification of PP2A complexes as described for Figure 1. (**B**) AML patients were analyzed for PP2A activity according to their FLT3 mutational status. (**C**–**E**) Quantitation of immunoblot analysis of mononuclear cells isolated from AML patients for (C) pY307-PP2Ac relative to total PP2A-C; (D) total PP2A-C; and (E) PP2A-A expression values were determined by dividing the densitometric volume of the test band by that of β-actin, and then normalized to the value for BaF3 cells which were run as a positive control on all gels. Mann-Whitney rank sum test *p < 0.05 compared to NBM (A) or WT-FLT3 patients (B–E). (**F** and **G**) AML patient BM mononuclear cells were treated with F) FTY720 (0–10 μM) or (G) AAL(S) (0–10 μM) for 24 h and percent viability determined by annexin V/PI negativity, and the IC50 calculated by spline regression. Each dot represents an individual patient; bar shows the median. (**H** and **I**) The IC50 for H) FTY720 or I) AAL(S) in human mononuclear cells determined by annexin V/PI negativity at 24 hr +/− 50 ng/ml FL. Matched patients are shown with a connecting line. (**J**) AML patient BM mononuclear cells were treated with or without 1 μM FTY720 for 12 h, and the PP2A activity measured as above. Each patient was normalized to their own untreated activity value to gain a fold change in activity. This was then graphed against the IC50 for FTY720 as determined in (F), followed by linear regression analysis.

To examine the clinical relevance of FLT3-ITD induced PP2A inhibition, we determined the activity of PP2A in bone marrow (BM) derived mononuclear cells isolated from 26 primary AML patients (Supplementary Table S1). AML patient samples exhibited lower PP2A activity compared to BM mononuclear cells isolated from healthy donors (NBM 4.60 ± 0.3 v's AML 2.83 ± 0.2 PO_4_/μg, *p* = 0.015) (Figure 3A). Blasts isolated from patients expressing FLT3-ITD displayed lower PP2A activity (2.28 ± 0.4 PO_4_/μg) than those expressing WT-FLT3 (3.43 ± 0.3 PO_4_/μg; *p* = 0.011) (Figure 3B). FLT3-D835^+^ blasts also displayed lower PP2A activity (2.57 ± 0.84 PO_4_/μg; *p* = 0.15) than WT-FLT3, but this was not statistically significant. Phosphorylation of the PP2A catalytic subunit at Tyrosine-307 is a marker of inactive PP2A, and consistent with the activity assays, immunoblotting revealed significantly higher levels of pPP2A-C^Y307^/PP2A-C in FLT3-ITD and FLT3-D835^+^blasts, compared to WT-FLT3 blasts (Figure 3C; Supplementary Figure S4A). No significant changes were observed in total PP2A-C levels (Figure 3D), however expression of the structural PP2A-A subunit was lower in the in FLT3-ITD+ AML patient mononuclear cells, compared to WT-FLT3 cells (Figure 3E; Supplementary Figure S4A). Thus FLT3-ITD^+^ AML patients have lower PP2A activity, higher pY307-PP2Ac, and lower PP2A-A expression than WT-FLT3 patients, suggesting that PP2A inhibition may be clinically important in FLT3-ITD^+^ AML.

A trend towards decreased PP2A-B56α, -B56δ and B′α 130 kD protein was also observed in FLT3-mutant AML blasts compared to WT-FLT3 blasts (Supplementary Figure S4B). In addition, analysis of publically available RNAseq and microarray data in the TCGA database [35] revealed that PP2A genes B55δ (PPP2RD), B56δ (PPP2R5E) and B′α (PPP2R3A) were significantly lower in FLT3-ITD+ patients compared to WT-FLT3 patients (Supplementary Figure S5).

### FTY720 induces cell death of primary human AML blasts

Primary human AML blasts were treated with FTY720 or AAL(S) (1–10 μM; 24 h) and cell death was assessed by Annexin V/PI staining. All AML samples displayed some sensitivity to FTY720, with a median IC50 of 3.1 μM (range 0.5–8.7) (Figure 3F). The median IC50 was lower in FLT3-ITD^+^ blasts (2.4 μM) than WT blasts (3.33 μM) but this was not statistically significant (Figure 3F). AML blasts were also sensitive to AAL(S) (median IC50 3.1 μM; range 5.1–4.7 μM) with no significant difference between WT and FLT3-ITD patients (Figure 3G). We further examined if the addition of exogenous FL affected the sensitivity to PP2A activators. For most patients blasts the addition of exogenous FL slightly reduced the IC50 to FTY720 (Figure 3H) and AAL(S) (Figure 3I), irrespective of their FLT3 status, but overall this was not statistically significant. It should be noted however that exogenous FL also had no significant effect on viability in any of the samples in the absence of drug (not shown).

For 4 patients we had sufficient material to test PP2A activity after treatment with 1 μM FTY720 for 12 hr. In cases where PP2A activity increased in response to FTY720 (i.e. positive fold induction), these cells were more sensitive to FTY720 induced apoptosis (i.e. lower IC50) (Figure 3J), suggesting that the ability for FTY720 to induce PP2A activity may determine *in vitro* drug sensitivity.

FTY720 also induced cell death in a purified population of CD34^+^/CD38^−^/CD123^+^ cells enriched for leukemic stem and progenitor cells (LSPCs) from FLT3-ITD^+^ AML patients (Supplementary Figure S6A, S6B), and was more effective than the TKIs AG1296 or CEP701 (Supplementary Figure S6C). In contrast, FTY720 or AAL(S) had no significant effect on long term self-renewal of normal CD34+ cells (Supplementary Figure S6D–S6G). Therefore PP2A activation may allow the selective targeting of AML blasts as well as LSPCs in FLT3^+^ patients.

### PP2A activators synergize with tyrosine kinase inhibitors

**Figure 4:**
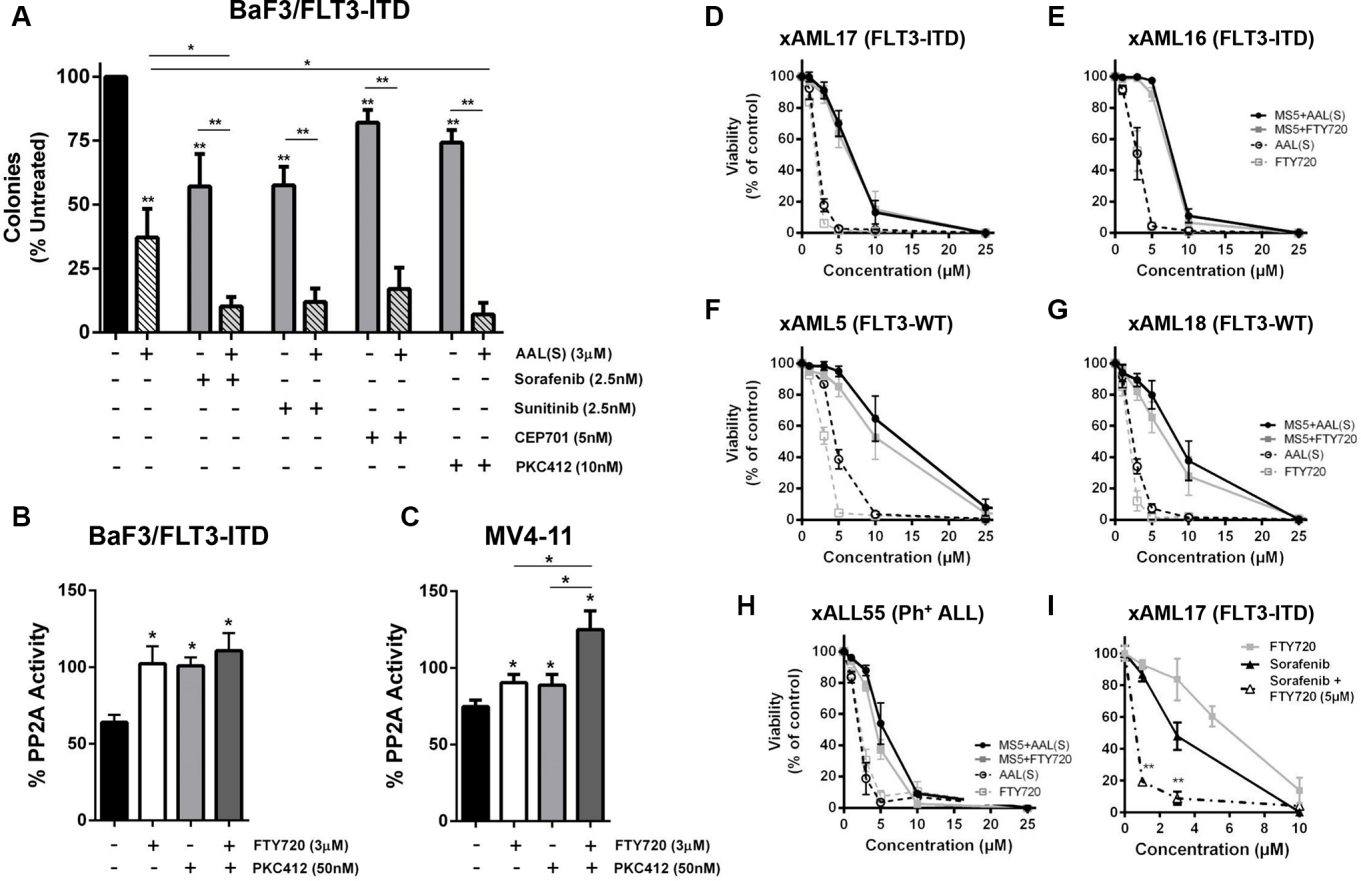
Combined effects of PP2A activation and FLT3 inhibitors (**A**) BaF3/FLT3-ITD cells were grown in methylcellulose medium for 7 days in the presence of AAL(S) ± Sorafenib, CEP701, PKC412 or Sunitinib. *Columns*, mean colony number (*n* = 3); bars, SEM. *p < 0.05, **p < 0.01, one-way ANOVA with Tukey's multiple comparison *t* test. (**B** and **C**) PP2A activity was measured in (B) BaF3/FLT3-ITD or (C) MV4-11 cells after treatment with indicated concentrations of FTY720 +/− PKC412 for 12 hr. *Columns*, mean (*n* = 3); bars, SEM. *p < 0.05, **p < 0.01, Students *t* test compared to untreated cells. (**D**–**I**) Human AML or ALL mononuclear cells were expanded in immunodeficient mice prior to *in vitro* culture with or without the BM stromal cell line, MS5. Cells were treated with FTY720 or AAL(S) for 24 h and viable cells examined by Annexin V/7AAD staining and flow cytometry. Leukemia cells were gated from MS5 cells based on size, hCD45+, and percent viability of leukemia cells shown as the percentage of Annexin V and 7AAD negative cells divided by the total number of leukemia cells. (D) xAML-17 (FLT3-ITD); (E) xAML-16 (FLT3-ITD); F) xAML-5 (FLT3-WT); (G) xAML-18 (FLT3-WT); H) xALL-55 (Ph^+^ ALL). I) xAML-17 (FLT3-ITD) in co-culture with MS5 was treated with FTY720, Sorafenib, or FTY720 + Sorafenib at indicated concentrations for 24 h and viable cells examined by Annexin V/7AAD as above. *indicates synergism according to the method of Webb [63].

We next tested the effects of PP2A activators in combination with FLT3 inhibitors. Treatment of BaF3/FLT3-ITD or MV4-11 cells with CEP701, PKC412, sunitinib, sorafenib or AC220, in combination with FTY720 or AAL(S), induced a greater effect than either drug alone, as assessed by resazurin assays. The combination index (CI) revealed additive, and in most cases synergistic effects in both cell lines (Table 2). As AAL(S) alone showed substantial inhibition of clonogenicity in the BaF3/FLT3-ITD cells, we further assessed AAL(S) together with TKIs in clonogenic assays. The combination of AAL(S) with sunitinib, CEP701, PKC412 or sorafenib induced a significant reduction in colony formation compared to treatment with the TKI alone (Figure 4A). Importantly, the combination of FTY720 or AAL(S) with kinase inhibitors had no effect on normal CD34+ bone marrow cells (Supplementary Figure S6F–S6G).

**Table 2:** Synergy of tyrosine kinase inhibitors and PP2A activators in FLT3-ITD cells

Combination Index (CI)^§^				
Drug Combination	BaF3/FLT3-ITD		MV4-11	
FTY720 + Sorafenib	0.79	++	0.47	+++
FTY720 + Sunitinib	0.92	±	0.31	+++
FTY720 + CEP701	0.90	±	0.27	+++
FTY720 + PKC412	0.80	++	0.35	+++
FTY720 + AC220	0.73	++	0.50	+++
AAL(S) + Sorafenib	1.11	−	0.51	+++
AAL(S) + Sunitinib	0.65	+++	0.41	+++
AAL(S) + CEP701	0.58	+++	0.42	+++
AAL(S) + PKC412	0.52	+++	0.49	+++
AAL(S) + AC220	0.65	+++	0.75	++

§ Combination index was calculated from the ED75 using Chou-Talalay analysis in the CalcuSyn software; -,antagonism (> 1.1); ±, additive (0.9–1.1); ++, moderate synergism (0.7–0.9); +++, synergism (0.3–0.7).

Given we showed that pharmacological inhibition of FLT3 could increase PP2A activity (Figure 2B, 2C), we next sought to determine if the synergism observed with TKIs and PP2A activators was associated with even higher PP2A activity. BaF3/FLT3-ITD and MV4-11 cells were treated with FTY720, PKC412, or both drugs for 12 hr. The combination slightly increased PP2A activity in the BaF3/FLT3-ITD cells (Figure 4B), and significantly enhanced activity in the MV4-11 cells, compared to either compound alone (Figure 4C). This suggests that the synergism observed in these cells is at least partly due to heightened PP2A activity levels.

### PP2A activators induce cell death of AML cells in co-culture with bone marrow stromal cells

The BM microenvironment provides significant protection for AML cells against chemotherapeutics [36]. To determine if PP2A activators can target AML cells protected by BM stromal cells, we utilized human AML blasts that had been expanded in NOD/SCID or NSG mice [37]. Isolated blasts were cultured with the mouse BM stromal cell line MS5. MS5 cells provided substantial protection of AML cells from FTY720 and AAL(S), however, both compounds could still induce cell death in a dose (Figure 4D–4I) and time (Supplementary Figure S7) dependent manner. FLT3-ITD^+^ AML cells were more sensitive to PP2A activators compared to WT-FLT3 AML cells in co-culture (Figure 4D–4G; Supplementary Table S2). A Ph^+^ ALL sample that is responsive to FTY720 *in vivo* [38] was used as a positive control in these experiments, and was the most sensitive to both FTY720 and AAL(S) (Figure 4H; Supplementary Table S2). We further tested the combination of a TKI and PP2A activator in a FLT3-ITD^+^ sample. A synergistic effect was observed for 5 μM FTY720 with 1nM or 3nM sorafenib in the FLT3-ITD^+^ xAML-17 (Figure 4I). This data suggests that PP2A activators may be efficacious in the *in vivo* setting, particularly in combination with TKIs.

## DISCUSSION

Activating mutations in FLT3 are the most common genetic aberration observed in AML and are associated with poor prognosis [7]. This study provides the first molecular link between activation of the FLT3 receptor and the tumour suppressor protein, PP2A. We have shown in cell lines and primary human AML blasts that oncogenic FLT3 signaling significantly suppresses PP2A activity, in association with decreased expression of the PP2A-A scaffolding and regulatory B subunits. Importantly, functional re-activation of PP2A using two independent compounds, FTY720 and AAL(S), inhibited growth and colony formation, and induced cell death in cells expressing FLT3-ITD. Of important clinical relevance, combined administration of FTY720 or AAL(S) together with a FLT3 inhibitor resulted in synergistic growth inhibition. Thus, specific activation of PP2A in concert with currently available kinase inhibitors may provide a unique approach for therapeutic targeting of AML patients expressing mutant FLT3.

FTY720 is an immunomodulatory agent in use as an oral therapy for multiple sclerosis. FTY720 is metabolized by sphingosine kinase-2 to FTY720-P, which targets the S1P receptors for degradation, leading to inhibition of lymphocyte trafficking [30, 39]. FTY720 has been proposed as an anti-cancer agent [18, 40], however its efficacy may be limited with clinical toxicities including transient bradycardia, macular oedema, and brain inflammation, thought to be due to the effects of FTY720-P on sphingosine-1-phosphate receptors [41, 42]. Furthermore, FTY720-P itself may have pro-proliferative properties [30, 39]. FTY720 analogues that are not targets for phosphorylation by sphingosine kinase-2, such as AAL(S), may have fewer toxicities and be more useful anti-cancer drugs [43]. AAL(S) was more effective at colony inhibition than FTY720. Consistent with this notion, another non-phosphorylatable FTY720 analogue, OSU-2S, was more effective than FTY720 in mouse models of hepatic cellular carcinoma [44], and showed efficacy against human CML stem cells [34]. Further preclinical testing of non-phosphorylatable FTY720 analogues in both hematopoietic and solid tumors is therefore warranted. Importantly, we show here that FTY720 and AAL(S) had no effect on the survival of normal CD34+ cells, an important consideration for clinical application.

We found that PP2A activators exhibited synergistic effects with TKIs. In support of this OP449, a peptide inhibitor of SET that induces PP2A activation, was recently shown to synergise with the FLT3 inhibitor AC220 in FLT3-ITD^+^ MOLM-14 cells [45]. OP449 also displayed synergy with JAK Inhibitor I in a JAK3 mutant AML cell line, CMK, and with Ara-C in the NRAS mutant acute promyelocytic cell line HL-60 [45]. Additive effects of a chemically distinct PP2A activator, forskolin, with Ara-C and Idarubicin have also been reported in the KG-1 and HEL AML cell lines [24]. Therefore PP2A activation, either via sphingosine analogues or direct SET inhibitors, in combination with TKIs and/or standard chemotherapy, is a potential therapeutic strategy for AML. While additive effects would be expected given both strategies ultimately target similar pathways, the mechanism for the observed synergism remains unclear. A recent study reported that Pim kinases exert proximal control of FLT3-ITD signalling, and inhibition of Pim kinases was synergistic with FLT3 inhibitors [46]. Given that PP2A induces degradation and inactivation of Pim-1 [47], one possibility is that enhancing PP2A activity with FTY720 or AAL(S) results in Pim-1 inhibition, hence facilitating the activity of TKIs against FLT3-ITD.

We found that the intrinsic phosphatase activity of PP2A was significantly lower in AML blasts compared to mononuclear cells isolated from healthy controls. This is in agreement with a previous study using PP2A-C^Y307^ hyperphosphorylation as a measure of PP2A activity [24]. We further demonstrate that AML patients expressing FLT3-ITD have significantly lower PP2A activity than WT-FLT3 patients. Both the BaF3/FLT3-ITD cells and primary FLT3-ITD^+^ AML cells displayed reduced expression of the structural PP2A-A subunit. We have previously reported reduced PP2A-A expression in mutant c-KIT myeloid cells, and overexpression of PP2A-A inhibited cell growth and induced apoptosis [23]. Thus, downregulation of PP2A-A may be a common mechanism utilized by oncogenic tyrosine kinases to drive leukemia. PP2A-A knockdown has been shown to induce a concomitant loss of PP2A-B55, -B56α and –B56δ subunit proteins, and reduced PP2A activity [48, 49], therefore, the decreased PP2A-A may also contribute to the downregulation of PP2A-B subunits we observed in the FLT3-ITD cells. Analysis of the TCGA database also showed reduced gene expression of several PP2A regulatory B subunits in FLT3-ITD^+^ compared to WT-FLT3 AML patients, therefore multiple mechanisms of reduced PP2A protein expression are likely involved in AML. Regardless, reduced PP2A expression appears to be a common event in AML [24, 50] and has been repeatedly shown to contribute to oncogenesis (e.g. see reviews [18, 51–54]). For example, loss of PP2A-B56α results in accumulation of c-MYC [55], while reduced PP2A-B55α leads to enhanced phosphorylation of AKT [56, 57], both important downstream effectors of FLT3.

Pharmacological re-activation of PP2A with FTY720 reduced phosphorylation of AKT^S473^ and ERK1/2^T202/Y204^, suggesting that FTY720-induced cell death and growth inhibition is due in part to inactivation of these pathways. FTY720 also inhibited JAK2 and its downstream target STAT5 in the BAF3/FLT3-ITD cells, but not in the human MV4-11 cells, thus the relevance of this pathway requires further investigation. Interestingly however, while JAK2 has previously been shown to inhibit PP2A [34], we found that PP2A inhibition in our cells was not dependent on active JAK2.

In summary, our data shows that activation of FLT3 inhibits the activity of PP2A. Importantly, chemical activators of PP2A can successfully overcome this inhibition and suppress the growth and survival of AML cells signaling through FLT3, suggesting PP2A as a therapeutic target in FLT3^+^ AML, and adding further weight to the case for clinical trials of PP2A activators in myeloid leukemias. Taken together with previous work, this study highlights the interplay between PP2A and oncogenic kinases including BCR-ABL [22], c-KIT [23] and FLT3, indicating that functional inactivation of PP2A may represent a crucial event in the initiation and conservation of leukemia growth and survival, and likely other cancers driven by oncogenic activation of tyrosine kinases.

## MATERIALS AND METHODS

### Drugs

FTY720, CEP701, PKC412 and okadaic acid (OA) were from Cayman Chemicals; sorafenib, sunitinib and ruxolitinib from Selleckchem; AC220 from LC Laboratories; and AAL(S) was synthesized as previously described [58]. The chemical structures of all drugs are shown in Supplementary Figure S8.

### Cell lines and patient samples

Murine pro-B BaF3 cells were maintained in RPMI 1640 containing 10% fetal calf serum (FCS), 2 mM L-glutamine, 25 mM HEPES and 4 ng/ml murine IL-3 (BioLegend). BaF3 parental cells were stably transduced with empty vector (EV), WT, D835V, D835Y or ITD forms of human FLT3 by retroviral transduction. BaF3/EV cells were maintained in 4 ng/ml IL-3, WT maintained in 4 ng/ml IL-3 or 50 ng/ml FLT3 ligand (FL) and the mutant FLT3 cells maintained without growth factor. The FDC.P1 WT-FLT3 cells [33] were maintained in 25 units/ml GM-CSF (GM) or 50 ng/ml FL. MV4-11 cells, a human FLT3-ITD+ AML cell line derived from the peripheral blood of a 10 year old male, were initially established by Lange et al. [59] and purchased from the ATCC. MV4-11 cells were maintained in RPMI 1640 with 10% FCS and 2 mM L-glutamine. Cell lines were routinely screened for authenticity by the Australian Genome Research Facility.

Human bone marrow (BM) samples were obtained from AML patients according to institutional guidelines as previously described [60] (Supplementary information). Normal CD34+ BM mononuclear cells were purchased from Stemcell Technologies or Lonza.

### Co-culture assays

The mouse BM mesenchymal stromal cell-line MS5 was kindly provided by Prof. Mori (Niigata University, Japan) [61]. MS5 cells (10^4^) were grown to confluence in 96 well U-bottom plates in αMEM supplemented with 10% FCS, penicillin/streptomycin, and L-glutamine. Human AML xenograft cells (5 × 10^4^) were added to the confluent MS5 monolayer in IMDM with 0.5% FCS, 1% penicillin/streptomycin and L-glutamine. FTY720 and AAL(S) (0–25 μM) were added for 24–48 h. Cells were harvested with trypsin, stained with Annexin V-PE (BD Biosciences, CA, USA), an anti-human CD45-APC antibody, and 7AAD (BioLegend, CA, USA), and enumerated on a FACSCalibur flow cytometer (BD Biosciences). Cells positive for CD45, and negative for Annexin V and 7AAD were considered viable leukemic cells.

### PP2A activity, immunoprecipitation and immunoblotting

PP2A-C immunoprecipitates were isolated with the Precipitor^™^ magnetic bead based platform (Abnova) using PP2A-C 1D6 mAb (Santa Cruz Biotechnology, SC25564) and phosphatase activity against a phosphopeptide (KRpTIRR) was determined as previously described [23]. FLT3 immunoprecipitation [33] and western blotting [23] was performed as previously described (see Supplementary Material for more details).

### Cell proliferation, cell death, and clonogenic assays

Cell viability was determined using a resazurin assay as previously described [23]. For combination assays cells were treated at fixed drug ratios and the combination index calculated according to Chou-Talalay [62] using CalcuSyn software (Biosoft, Ferguson, MO, USA). Colony formation in methylcellulose, and cell death measured using the Annexin-V FITC apoptosis detection kit (BD Biosciences), were performed as previously described [23].

## SUPPLEMENTARY MATERIALS



## Abbreviations

AML: Acute myeloid leukemia
PP2A: Protein phosphatase 2A
FLT3: Fms-like tyrosine kinase 3
ALL: Acute lymphoid leukemia
CML: Chronic myeloid leukemia
ITD: Internal tandem duplication
FL: FLT3 ligand
EV: Empty vector
TKI: Tyrosine kinase inhibitor
WT: Wild type
