# Supplementary material for: Activation of protein phosphatase 2A in FLT3+ acute myeloid leukemia cells enhances the cytotoxicity of FLT3 tyrosine kinase inhibitors
Source: Oncotarget. 2016 Jun 18;7(30):47465–78. doi: 10.18632/oncotarget.10167 (PMC5216954; doi:10.18632/oncotarget.10167)
Supplement: Supplementary file 1 [file oncotarget-07-47465-s001.pdf]

Activation of protein phosphatase 2A in FLT3+ acute myeloid leukemia cells enhances the cytotoxicity of FLT3 tyrosine kinase inhibitors

Supplementary Materials

SUPPLEMENTARY METHODS

Human mononuclear cells

Human bone marrow samples were obtained from AML patients according to institutional guidelines as previously described [28]. Studies were approved by human ethics committees of the Royal Adelaide Hospital, the University of Newcastle, the South Eastern Sydney and Illawarra Area Health Service, and the Hunter New England Area Health. Mononuclear cells were isolated by Ficoll-Hypaque density-gradient centrifugation, washed, and resuspended at 5×10^5/mL in IMDM, 0.5% FCS, 1% penicillin/streptomycin. In some experiments cells were cultured in the presence of 50 ng/ml FL as indicated. All samples had a minimum of 75% AML blasts. FLT3 status was determined using routine pathology, and was confirmed by genomic DNA sequencing utilizing juxtamembrane domain (Forward- 5′-GCAATTTAGGTATGAAAGCCAGC-3′, Reverse- 5′-CTTTCAGCATTTTGACGGCAACC-3′) and kinase domain (Forward- 5′-TTCACAGAGACCTGGCCG-3′, Reverse- 5′-GCCCAAGGACAGATGTGATG-3′) specific primers and sequenced by Sanger sequencing (AGRF; Brisbane, Australia). FACS purification of primary human $CD34^+CD38^-CD123^+$ leukemic stem and progenitor cells (LSPCs) was performed as previously described [28].

For primary human $CD34^+$ bone marrow cells, 500 cells were seeded in duplicate into 6-well culture plates with 1.1 ml methocult (StemCell Technologies, #H4034) in 0, 1 or 3 µM FTY720 or AAL(S). Total colonies were counted after 7 days and differential colonies counted after 14 days according to manufacturer's instructions.

Antibodies used for immunoblotting

The following primary antibodies were used for immunoblotting: anti-PP2A-A (Calbiochem); anti–PP2A-B56α, anti–PP2A-B56γ3, anti–PP2A-B56δ, and anti–PP2A-B56ε (Novus Biochemicals); anti-PP2Ac^{Y307} (Epitomics), anti-I1PP2A, anti-I2PP2A (SET; clone H-120), anti-FLT3 (Santa Cruz Biotechnology), anti-pAkt^{T308}, anti-pERK1/2$^{T202/Y204}$, anti-pMEK^{S221}, anti-pp38MAPK$^{T180/Y182}$, anti-pSTAT5^{Y694}, anti-pJAK2^{Y221}, anti-AKT, anti-ERK1/2, anti-MEK, anti-p38MAPK and anti-STAT5 (Cell Signaling Technology, Inc.); and anti-β-actin (ACTB) (Sigma Aldrich). Affinity-purified rabbit antibodies against PP2A-B55α[30] and PP2A-C[31] were previously described. Pixel densitometry volume quantification was determined using Multi Gauge software.

Statistical analysis

Statistical significance of differences between samples was assessed using a Student's *t*-test. Values shown are the mean ± SEM. All analyses were performed using GraphPad Prism 5 software (GraphPad Software Inc, CA, USA). IC_{50} values were calculated using cubic spline regression analysis using GraphPad Prism 5.0. For co-culture combination assays, synergy was determined by the Fractional Product method, calculated according to Webb[30].

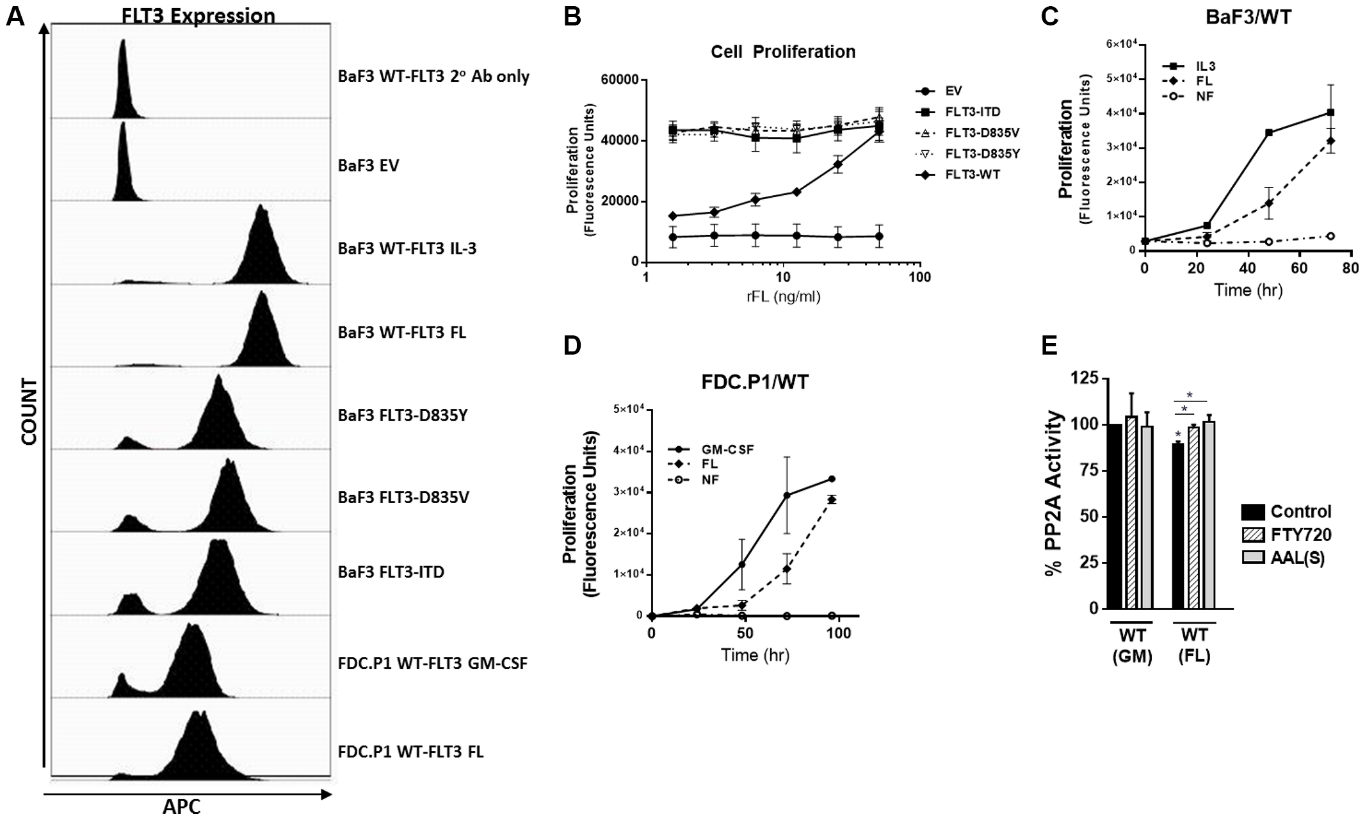

Supplementary Figure S1: Expression, proliferation and PP2A activity of FLT3 transduced BaF3 and FDC.P1 cells. (**A**) FLT3 surface expression was monitored routinely using an anti-FLT3/FLT2 (SF1.340; Santa Cruz) antibody and an APC-conjugated secondary antibody and analyzed on a FACSCalibur flow cytometer (BD Biosciences). (**B**) BaF3 parental cells were stably transduced with empty vector (EV), WT, D835V, D835Y or ITD forms of human FLT3 and grown in the presence of a range of FLT3 ligand (rFL) concentrations for 48 hr. Cell proliferation was determined using a resazurin assay and expressed as fluorescence units. (**C**) BaF3 and (**D**) FDC.P1 cells transduced with human FLT3-WT were grown in the presence of IL3, GM-CSF or FL as indicated. Cell proliferation was assessed by seeding triplicate wells of a 96-well plate with 1×10^4 cells/well, and growth determined by resazurin reduction over time. (**E**) PP2A complexes were isolated from FDC.P1 mouse myeloid cells transduced with WT FLT3, and grown in the presence of granulocyte-macrophage-colony stimulating factor (GM) or FLT3 ligand (FL), treated with or without 3 μM FTY720 or AAL(S) for 12 h, using immunoprecipitation with an anti-PP2A-C antibody. PP2A activity was determined by incubating the isolated PP2A-C complex with a PP2A-specific phosphopeptide and measuring free phosphate release using a colorimetric assay. Activity was calculated as a percentage of control WT-GM cells *Columns*; mean, *bars*; SEM, *$p < 0.05$, compared to WT-GM or untreated cells, as indicated; Students *t* test.

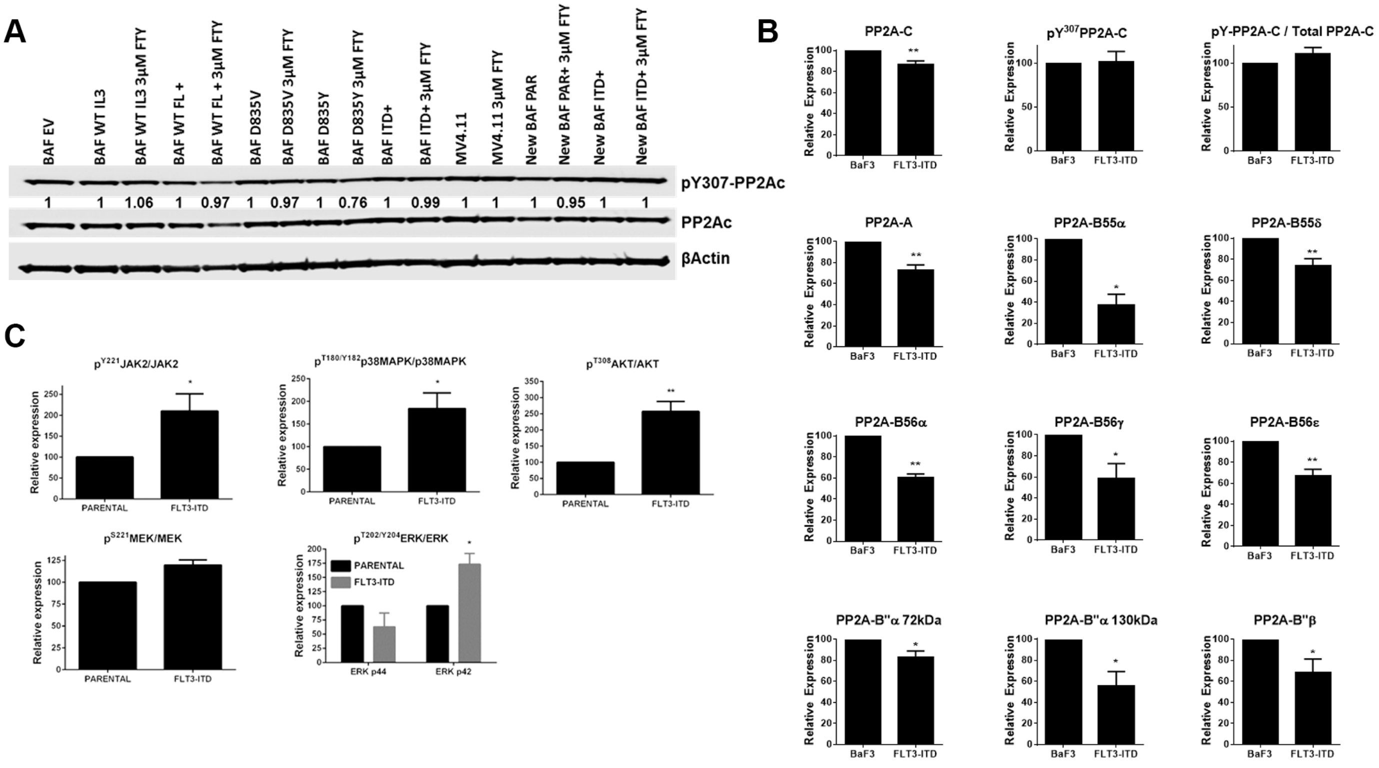

Supplementary Figure S2: (**A**) Immunoblot of BaF3 cells expressing an empty vector (EV) or FLT3, and FLT3-ITD$^+$ MV4-11 cells +/– 3 μM FTY720, 24 hr. Numbers underneath blot show the densitometric ratio of the pY307-PP2Ac band divided by the total PP2Ac band. β-Actin is shown as a loading control. (**B**) Quantitation of immunoblots of BaF3 parents and FLT3-ITD cells showing expression of pY307PP2A-C normalised to total PP2A-C, PP2A-C, PP2A-A, PP2A-B55α, -B55δ -B56α, -B56γ, -B56ε -B"α 130 kDa, –B"α 72 kDa,–B"β. β-actin was used as a loading control. Quantitation of densitometric volume relative to BaF3/Parental cells reveals a significant difference in several PP2A subunits. Columns, mean densitometric volume relative to β-actin normalised to BaF3 parental cells, $n = 4$; bars, SEM. *$p < 0.05$, **$p < 0.01$; Student's *t* test compared to BaF3. (**C**). Quantitation of immunoblots of BaF3 cell lysates showing expression of pJAK2(Y221)/JAK2, p-p38MAPK(T180/Y182)/p38MAPK, pERK(1/2)(T202/Y204)/ERK1/2), pMEK(S221)/MEK and pAKT(T308)/AKT. β-actin was used as a loading control. Quantitation of OD relative to BaF3/Parental reveals a significant difference in several signalling proteins. *Columns,* mean OD ($n = 4$) relative to β-actin normalised to parental; *bars*, SEM. *$p < 0.05$; **$p < 0.01$; Student's *t* test compared to parental.

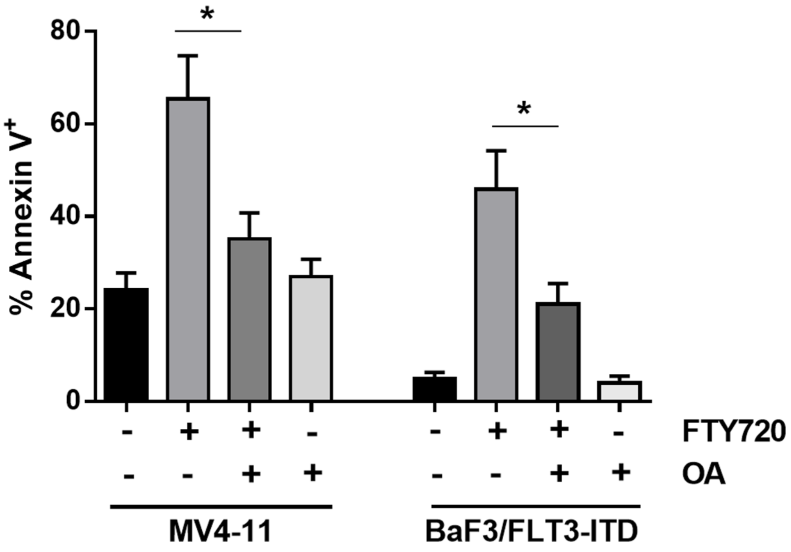

Supplementary Figure S3: PP2A activation is required for apoptosis induced by FTY720. MV4-11 cells were treated with 2 μM FTY720 +/– 25 nM OA (pretreatment for 2 hr + 24 hr with FTY720), and BaF3/FLT3-ITD cells treated with 3 μM FTY720 +/– 0.25 nM OA, for 24 hr and apoptotic cells determined by Annexin/PI assays. *Bars*, mean + SEM of at least 3 independent experiments performed in duplicate; *$p < 0.05$, between untreated and drug treated cells.

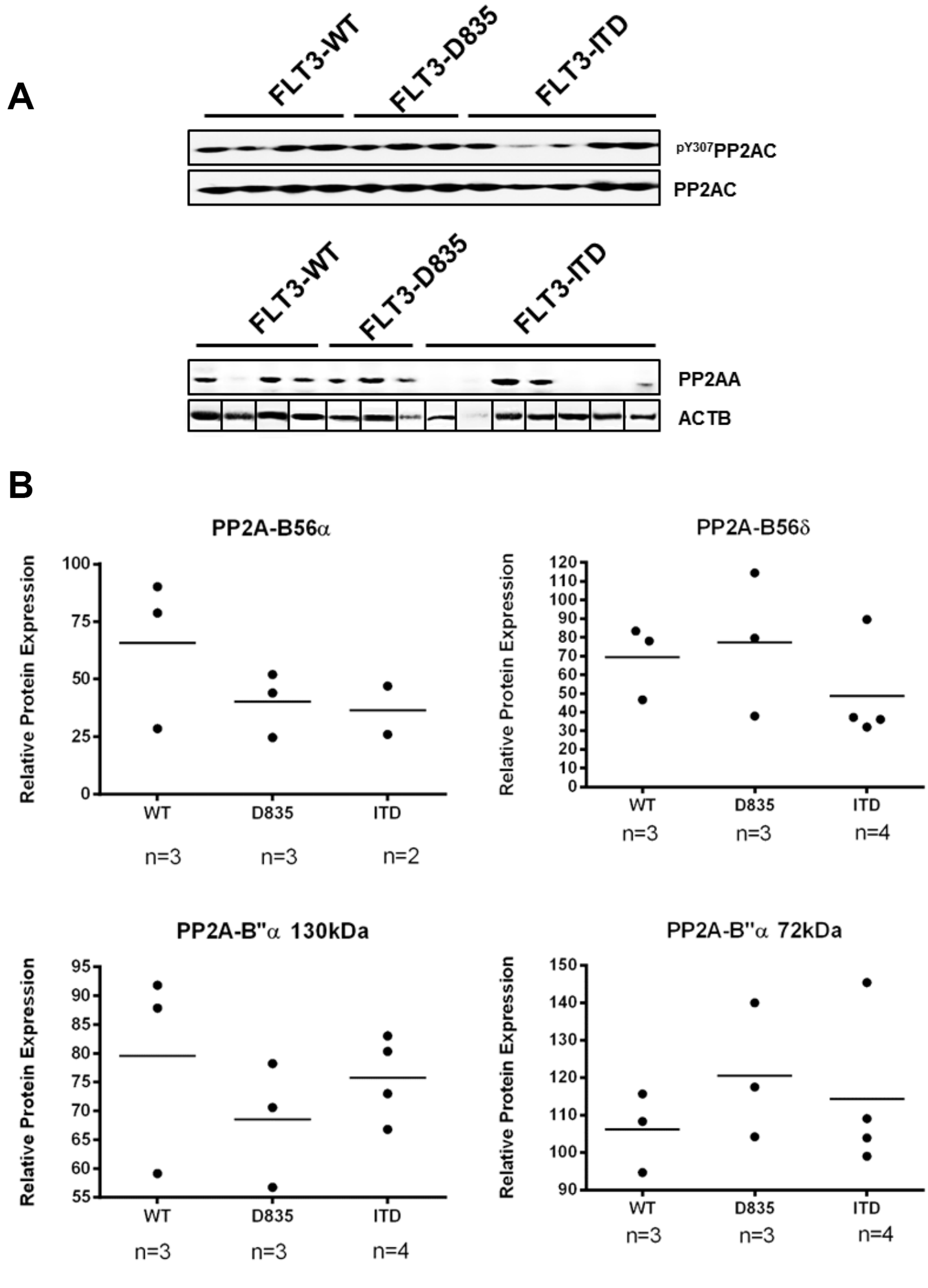

Supplementary Figure S4: PP2A subunit protein expression in WT and FLT3-mutant primary human AML mononuclear cells. (**A**) Bone marrow mononuclear cells were lysed and PP2A protein expression determined by immunoblot. Quantitation is shown in Figure 4 of the manuscript proper. The β-actin blot also included samples treated with drugs which were not included in this study, therefore was cropped to show only the untreated blasts. The black lines indicate where the image was cropped. (**B**) Bone marrow mononuclear cells were lysed and PP2A protein expression determined by immunoblot. Quantitation of densitometric volume of immunoreactive bands for PP2A-B56α, -B56δ, -B"α 130 kDa, and –B"α 72 kDa, was normalised relative to densitometric volume of the β-actin band.

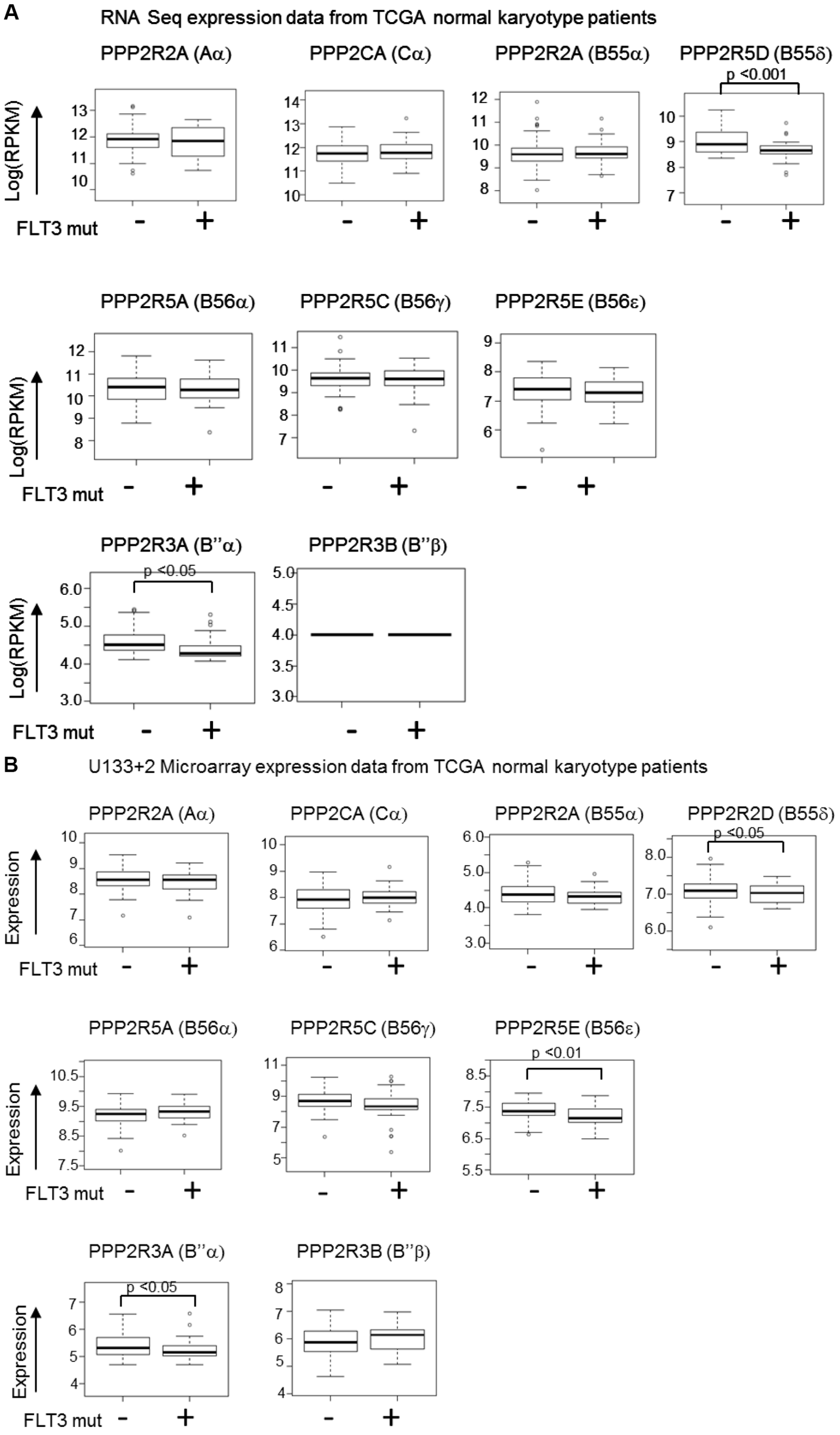

Supplementary Figure S5: Reduced gene expression of PP2A regulatory subunits in FLT3 mutant AML patients. (**B**) Gene expression data from U133+2.0 microarrays was normalised in R using the JustRMA() function in the affy library. For both datasets, the mean expression level of each PP2A subunit gene in FLT3 mutation positive samples was compared to non-mutated normal karyotype samples using a two-tailed *t*-test with $p < 0.05$. Box and whiskers plots show the median, first quartile and third quartile (box) and upper and lower limits of expression (whiskers) for each gene in FLT3 mutation negative and positive samples.

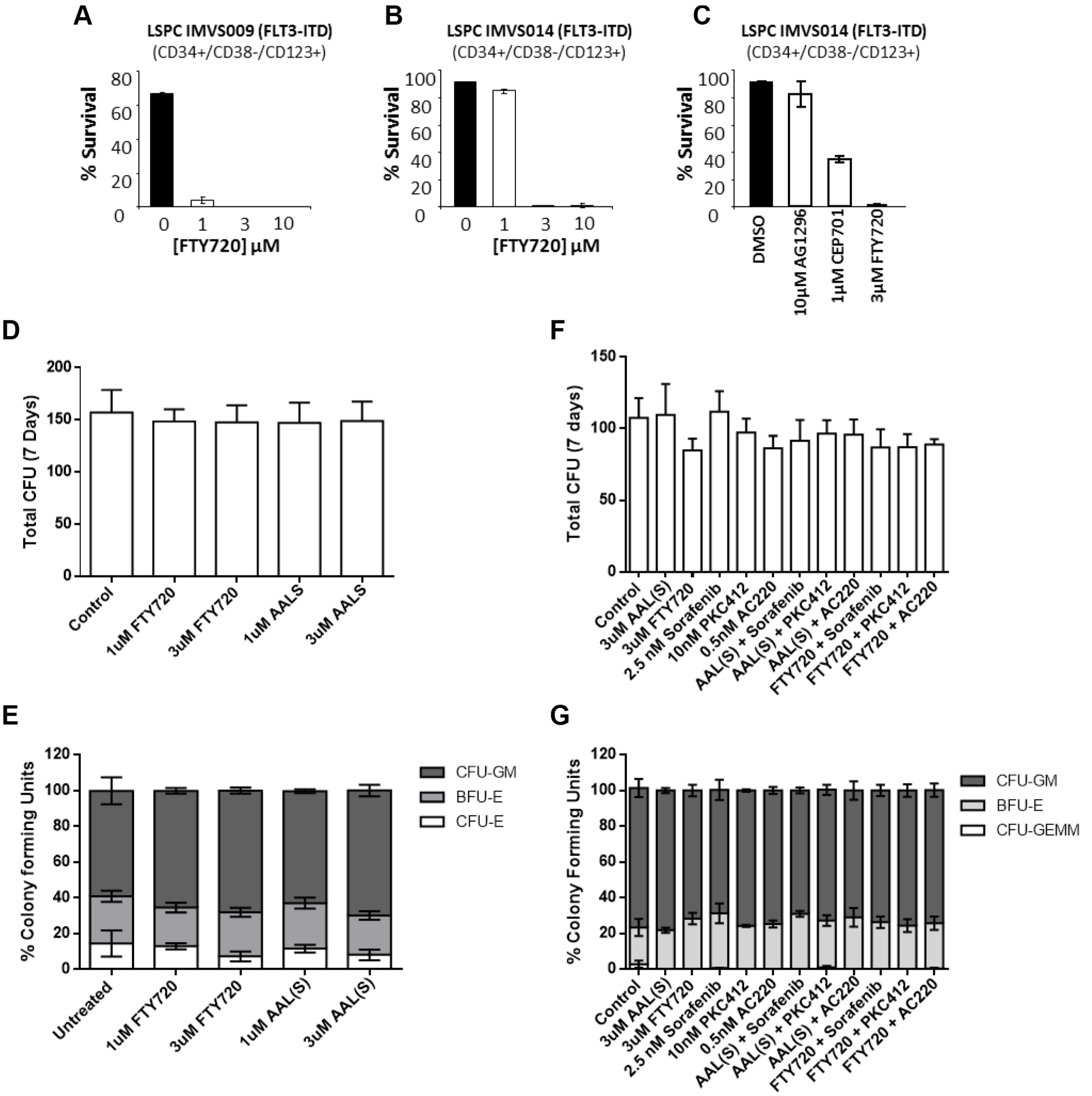

Supplementary Figure S6: Effect of PP2A on leukemia stem cells and normal CD34+ cells. (**A**–**C**) FACs purified LSPC were treated with FTY720, AG1296 or CEP701 for 48 hr. Survival is the percentage of annexin V negative cells. (**D**–**E**) Normal bone marrow CD34+ cells (n = 4–6) were obtained from StemCell Technologies. Cells were seeded in duplicate full cytokine complement methylcellulose and treated with FTY720 or AAL(S) at the indicated concentrations. (D) After 7 days total colony number was counted. (E) After 14 days differential colonies were scored and categorized as colony forming unit erythroid (CFU-E), burst forming unit erythroid (BFU-E), or colony forming unit granulocyte, macrophage (CFU-GM). (**F**–**G**) Normal human bone marrow CD34+ cells were purchased from Lonza (n = 2) and seeded in duplicate in full cytokine complement methylcellulose and treated with FTY720 or AAL(S) at the indicated concentrations. (F) After 7 days total colony number was counted. (G) After 14 days differential colonies were scored.Columns; mean, bars; SEM. There were no significant differences with any drug treatments compared to control untreated cells.

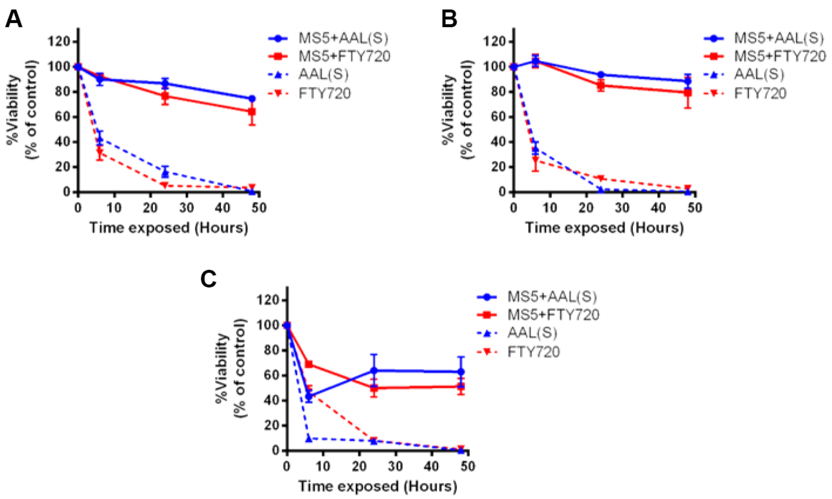

Supplementary Figure S7: Effects of FTY720 and AAL(S) over time on human leukemia in bone marrow stromal co-culture. Human leukemia cells were initially expanded in NOD-SCID mice prior to in vitro co-culture with the MS5 bone marrow stroma cell line. Cells were treated with 5 μM FTY720 or 5 μM AAL(S) and cell viability determined by Annexin V/7 AAD staining and flow cytometry at the indicated time points; *n* = 3. (**A**) xAML-17 (FLT3-ITD); (**B**) xAML-16 (FLT3-ITD); (**C**) ALL-55 (Ph+ ALL).

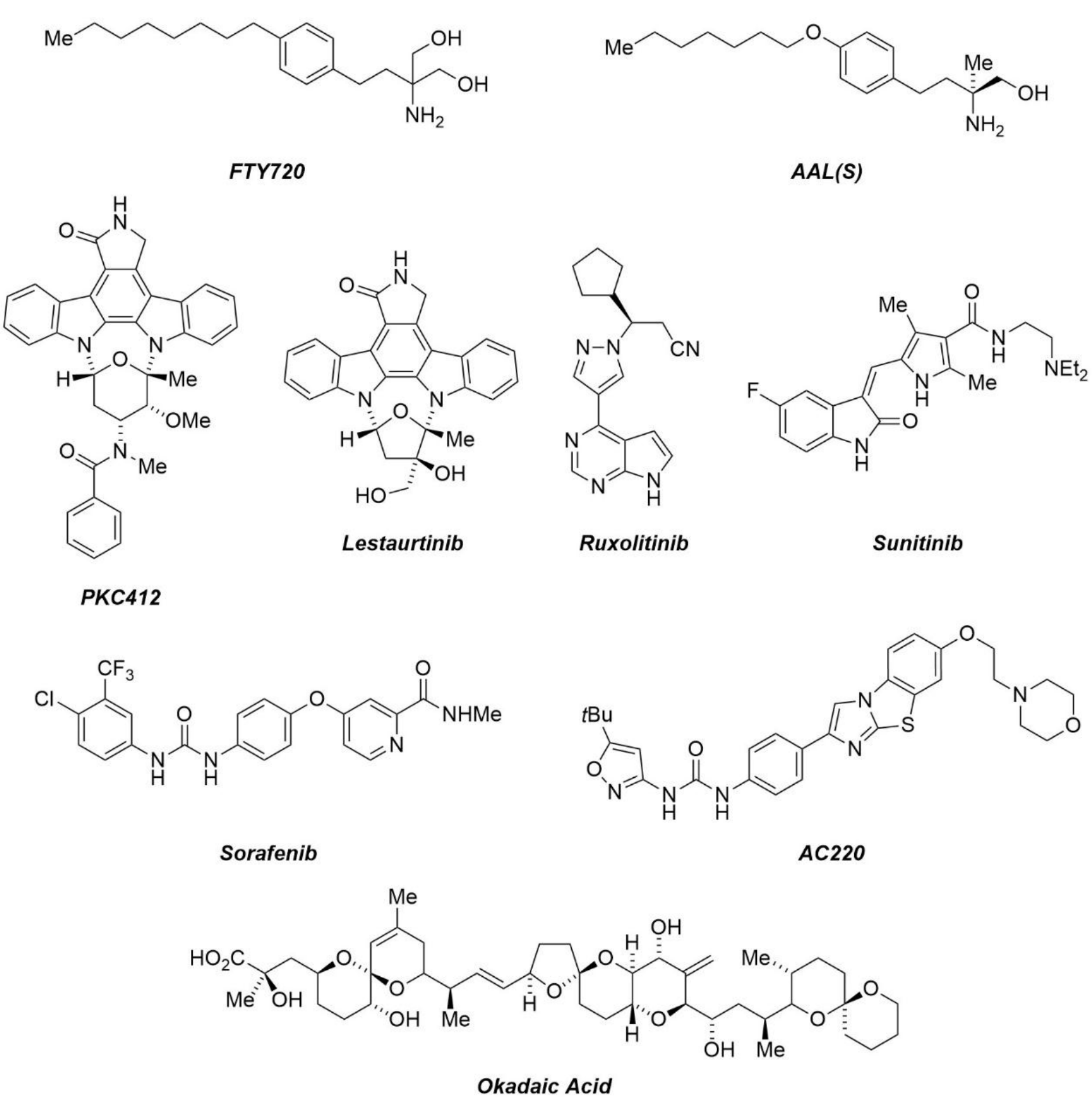

Supplementary Figure S8: Chemical structures of all drugs used in this study.

Supplementary Table S1: Patient Characteristics, PP2A activity and drug sensitivity

Patient ID	Sex	Age	FAB	Cytogenetics	FLT3 Status	PP2A Activity[I]	FTY720 IC50[II]	AALS IC50[II]
IMVS001	F	44	M1	MLL translocation, t(9:11)	WT	3.2	7.2	nd
IMVS002	F	62	M4	t(2;11)	WT	5.1	6.0	nd
IMVS003	F	48	M1	q 11	WT	3.5	nd	nd
IMVS005	M	72	M1	5q-	D835	0.9	nd	nd
IMVS006	M	70	M1	Normal	D835	3.7	3.7	nd
IMVS007	M	76	M5a	Trisomy 8	D835	3.1	3.4	nd
IMVS009	M	69	M1	Normal	ITD	2.7	2.6	nd
IMVS010	M	61	M4	Normal	ITD	0.8	nd	nd
IMVS011	M	67	M5	Normal	ITD	3.0	nd	nd
IMVS012	F	39	M1	Normal	ITD	1.1	nd	nd
IMVS013	F	76	M1	Normal	ITD	0.6	nd	nd
IMVS014	M	86	M4	Trisomy 8	ITD	3.6	2.6	nd
IMVS015	M	56	M4	Normal	ITD	3.6	nd	nd
IMVS016	F	55	M4	Normal	ITD	2.8	nd	nd
IMVS017	M	57	M1	Normal	ITD	3.0	2.2	nd
CBF001	F	67	M1	Normal	WT	2.1	5.5	nd
CBF003	F	73	M4	Normal	WT	3.4	5.4	nd
CBF007	F	67	M1	Relapse of CBF001	WT	3.0	nd	nd
CBF009	M	48	M4	Inv(16)(p13.1q2); CBFB-MYH11	WT	3.6	5.7	nd
CBF010	M	61	M5a	Complex karyotype including del 5q ; del 7 and 8;del 9p21	WT	2.3	nd	nd
CBF011	M	59	M1	t(9;11)(p22;123);MLLT3-MLL	WT	2.6	3.6	nd
CBF014	F	35	M0	trisomy 22 and del 18p	ITD	3.6	nd	nd
CBF016	F	63	M4	Normal	ITD	1.2	4.2	nd
CBF022	M	70	M2	Normal	WT	4.3	nd	nd
CBF023	F	68	M2	Normal	WT	Nd	4.7	nd
CBF024	M	56	M4	Normal	WT	3.6	nd	nd
CBF025	F	72	M1	del 7q	WT	4.4	nd	nd
CBF031	M	86	M1	Normal	WT	Nd	2.1	3.1
CBF036	F	78	M1	Normal	ITD	Nd	2.3	4.4
CBF039	M	52	M4/M5	t(10;11)(p12;q23); KMT2A-MLLT10	WT	Nd	2.7	3.8
CBF046	M	55	M1	Normal	WT	Nd	3.0	2.2
CBF047	F	52	M1	Normal	ITD	Nd	1.0	2.5
CBF048	M	65	M1	Normal	ITD	Nd	nd	2.7
CBF049	M	67	M2	45,X,-Y,t(8;21)(q22;q2) RUNX1-RUNX1T1	WT	Nd	3.8	3.1
CBF058	M	39	M4	Normal	ITD	nd	2.0	3.3
CBF065	M	43	M4	Normal	WT	nd	3.1	4.7
CBF066	M	52	M2	Normal	WT	nd	2.7	3.3
CBF067	M	41	M2	Normal	WT	nd	2.4	2.1
CBF008	F	44	NBM	Normal	NA	5.11	nd	nd
CBF026	M	64	NBM	Normal	NA	4.14	nd	nd
NBM1	M	56	NBM	Normal	NA	4.56	nd	nd

[I]PO4 released/µg protein; [II]Annexin V/PI, 24h drug treatment; nd, not determined; NA, not applicable; NBM, normal bone marrow.

Supplementary Table S2: IC50 of AML xenografts in bone marrow stromal co-culture

Sample	FLT3	FTY720 IC50[1]	AAL(S) IC50[1]
xAML-17	ITD	5.8 ± 0.5	6.7 ± 0.8
xAML-16	ITD	7.8 ± 0.2	8.3 ± 0.1
xAML-5	WT	12.8 ± 2.0	14.4 ± 2.3
xAML-18	WT	8.6 ± 2.0	10.2 ± 1.9
xALL-55 (Ph$^+$)	ND	4.5 ± 0.2	5.6 ± 0.8

[1]The IC50 (μM) was calculated using spline regression based on the % survival as determined by annexin V/7AAD assay using 0, 1, 3, 5, 10 μM FTY720 or AAL(S) for 24 h (Figure 6). Data represents mean ± SEM of at least 3 independent experiments each run in duplicate. ND, not determined.
